# Use of xylosidase 3C from *Segatella baroniae* to discriminate xylan non-reducing terminus substitution characteristics

**DOI:** 10.1186/s13104-024-06835-3

**Published:** 2024-06-24

**Authors:** Franz J. St John, Loreen Bynum, Dante A. Tauscheck, Casey Crooks

**Affiliations:** Institute for Microbial and Biochemical Technology, Forest Products Laboratory, USDA Forest Service, One Gifford Pinchot Dr, Madison, WI 53726 USA

**Keywords:** Glycoside hydrolase family 3, GH3, Xylosidase, *Segatella baroniae*, *Prevotella bryantii*, Xylooligosaccharides, XOS

## Abstract

**Objective:**

New characterized carbohydrate-active enzymes are needed for use as tools to discriminate complex carbohydrate structural features. Fungal glycoside hydrolase family 3 (GH3) β-xylosidases have been shown to be useful for the structural elucidation of glucuronic acid (GlcA) and arabinofuranose (Ara*f*) substituted oligoxylosides. A homolog of these GH3 fungal enzymes from the bacterium *Segatella baroniae* (basonym *Prevotella bryantii)*, Xyl3C, has been previously characterized, but those studies did not address important functional specificity features. In an interest to utilize this enzyme for laboratory methods intended to discriminate the structure of the non-reducing terminus of substituted xylooligosaccharides, we have further characterized this GH3 xylosidase.

**Results:**

In addition to verification of basic functional characteristics of this xylosidase we have determined its mode of action as it relates to non-reducing end xylose release from GlcA and Ara*f* substituted oligoxylosides. Xyl3C cleaves xylose from the non-reducing terminus of β-1,4-xylan until occurrence of a penultimate substituted xylose. If this substitution is O2 linked, then Xyl3C removes the non-reducing xylose to leave the substituted xylose as the new non-reducing terminus. However, if the substitution is O3 linked, Xyl3C does not hydrolyze, thus leaving the substitution one-xylose (penultimate) from the non-reducing terminus. Hence, Xyl3C enables discrimination between O2 and O3 linked substitutions on the xylose penultimate to the non-reducing end. These findings are contrasted using a homologous enzyme also from *S. baroniae*, Xyl3B, which is found to yield a penultimate substituted nonreducing terminus regardless of which GlcA or Ara*f* substitution exists.

**Supplementary Information:**

The online version contains supplementary material available at 10.1186/s13104-024-06835-3.

## Introduction

Xylan is the second most abundant polysaccharide among terrestrial plants. This polysaccharide is composed of β-1,4-linked xylose and is remarkable for the diversity of its xylan main-chain substitutions. These include the sugars 4-O-methyl-α-D-glucuronic acid (GlcA) and α-L-arabinofuranose (Ara*f*) and well as acetylation. GlcA substitutions are strictly linked through the O2 hydroxyl, while Ara*f* and acetyl groups can be found linked through the O2 and O3 hydroxyls of the xylose. The presence and the substitution nature of these appendages along the xylan chain (i.e. substitution frequency, random vs. periodic) is plant type and tissue dependent and results in many unique forms of xylan [1, 2].

Given this natural complexity, a variety of glycoside hydrolase (GH) enzymes have evolved for the complete degradation of xylans [3–5]. Arguably the most important type of GH involved in this process is an endo-β-1,4-xylanase (endoxylanase) which cleaves xylan internally to yield smaller xylan chains with eventual limit products consisting mostly of small neutral xylooligosaccharides and larger GlcA and/or Ara*f* substituted oligoxylosides [6, 7]. GlcA and Ara*f* debranching enzymes may act on the xylan main chain or be more specific for the substituted oligosaccharide endoxylanase hydrolysis products [8–10]. As xylooligosaccharides are generated, β-xylosidases liberate xylose from the non-reducing terminus which then may be directly assimilated by microorganisms. Additionally, numerous other unique xylan active enzymes have been reported in recent years [9, 11–17].

**Fig. 1:**
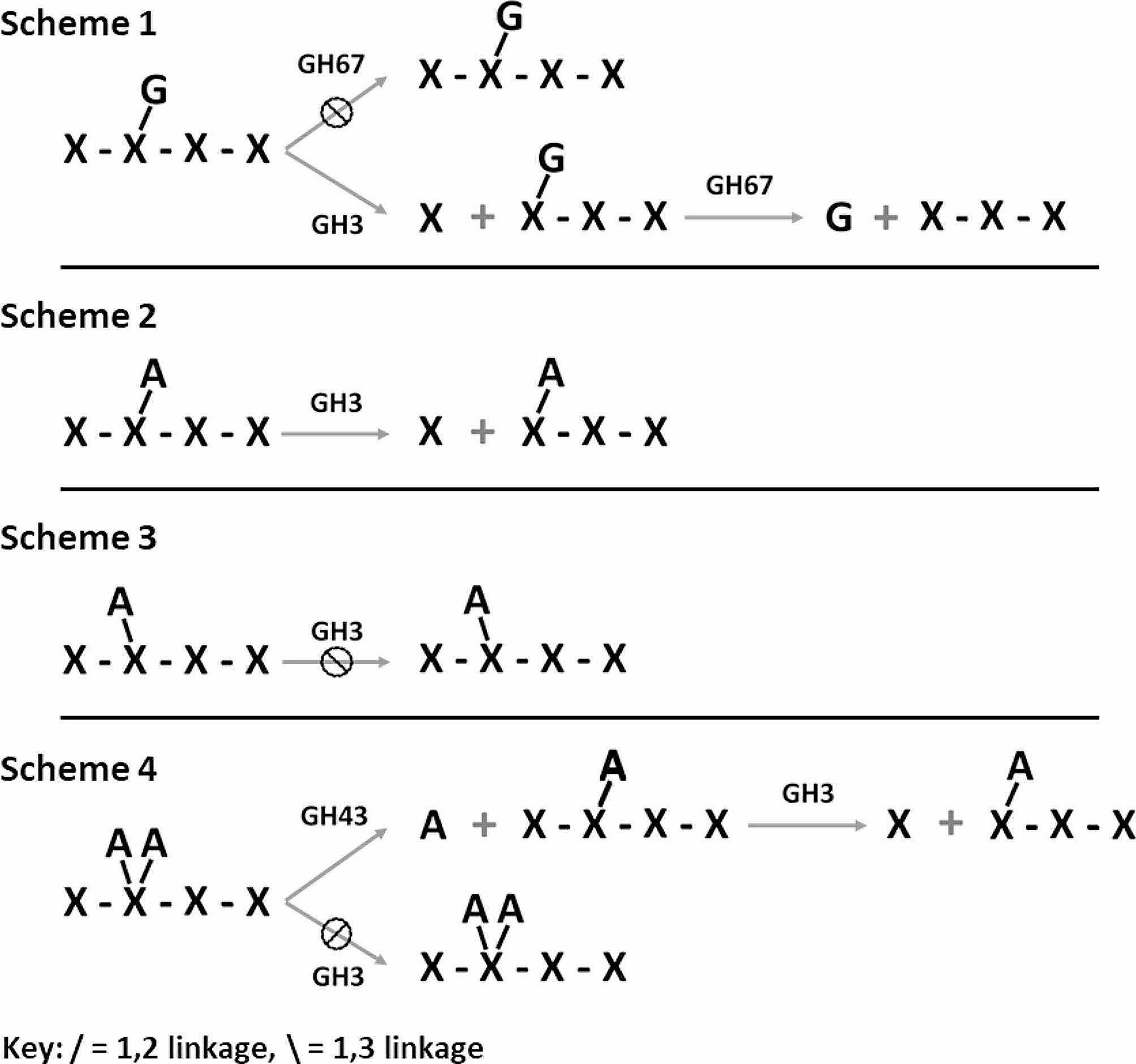
Schemes depicting xylooligosaccharide structure analysis based on selective enzymology. The aldouronate XUXX, which is the primary glucuronate substituted limit product of glucuronoxylan hydrolysis by GH11 endoxylanases [6], cannot be hydrolyzed with a GH67 alpha-glucuronidase until first being hydrolyzed with a GH3 β-xylosidase [21] that will remove the terminal xylose (scheme 1). The α-1,2 arabinofuranose substituted xylotetraose as shown in scheme 2 can be readily hydrolyzed by some GH3 β-xylosidases, while the isomer shown in scheme 3 bearing a α-1,3 arabinofuranose on the same xylose position cannot be hydrolyzed by some GH3 β-xylosidases [19], presumably due to the blocking of catalytic site access by the altered arabinose position. Lastly, as shown in scheme 4, the double substituted arabinofuranose can only be processed by the GH3 β-xylosidase if first treated with a specific GH43 arabinofuranosidase as shown in scheme 2

Biochemically characterized xylanases can be utilized as tools to elucidate the sequence/structure of the substituted xylooligosaccharides which result as major limit products of endoxylanases. This is of particular importance for the discovery of new endoxylanase families which have an unknown mode of action toward xylan. A GH67 α-glucuronidase is a good example, as it specifically liberates α-1,2-linked GlcA only when it is linked to the non-reducing terminal xylose (Fig. 1, Scheme 1) [18]. Some GH3 β-xylosidases can liberate xylose from the non-reducing terminus when the penultimate xylose is substituted on the O2 hydroxyl with either GlcA or Ara*f* (Fig. 1, Schemes 1 & 2). For these unique xylosidases an O3 Ara*f* on the same penultimate xylose prevents hydrolysis of the terminal unsubstituted xylose (Fig. 1, Scheme 3). With respect to this functional specificity, a *Trichoderma reesei* enzyme has been best characterized [6, 19, 20]. Although an *Aspergillus niger* xylosidase has once been reported to be used for oligoxyloside structure discrimination [21], the specific enzyme was not previously characterized. Research from our laboratory involving the purification, functional assessment and mass spectrophotometer peptide sequencing, confirmed that a GH3 xylosidase from *A. niger* N402 displays the previously reported activity (unpublished research). In this case, the sequenced peptides associated with the observed xylosidase function were most homologous with the XlnD (UniProt Accession No. A2QA27) from *A. niger* CBS 513.88. Perhaps the best example of an enzyme with highly specialized function, the GH43 arabinofuranosidase, AXH-D3 from *Bifidobacterium adolescentis* specifically hydrolyzes the O3 Ara*f* only from doubly Ara*f* substituted xyloses (Fig. 1, Scheme 4) [22, 23]. These enzymes have been utilized numerous times as biotechnological tools for xylooligosaccharide structure determination [7, 21, 24, 25]. Employing these and similar characterized enzymes facilitates new discoveries regarding xylan utilization.

A central capability of several of these important studies is the unique functional aspects of the GH3 β-xylosidase. Several GH families contain enzymes having xylosidase activity. However, not all xylosidases cleave a non-reducing terminal xylose when the penultimate xylose is substituted. Surprisingly, not even all GH3 xylosidases function in this same manner [26]. As the *T. reesei* [6] and *A. niger* [21] GH3 xylosidases were not commercially available, we sought to characterize a bacterial version for routine laboratory studies. A GH3 β-xylosidase from *Segatella baroniae* (basonym *Prevotella bryantii)*, Xyl3C, based on an original report [26] appeared possibly to have the desired functionality although this potential was not established in that study. In the current report, we have cloned, expressed, and purified the Xyl3B and Xyl3C enzymes from *S. baroniae* and performed studies with Xyl3C to confirm that this β-xylosidase has the functionality to allow it to be utilized for substituted xylooligosaccharide structure elucidation.

## Methods

### Biochemicals and reagents

Xylobiose (X_2_), xylotriose (X_3_), xylotetraose (X_4_), xylopentaose (X_5_), xylohexaose (X_6_), 4-nitrophenyl-β-D-xylopyranoside (pNP-Xyl), 4-nitrophenyl-β-D-glucopyranoside (pNP-Glu), 4-nitrophenyl-α-L-arabinopyranoside (pNP-Ara*p*), the Ara*f* substituted xylooligosaccharide (XOS) 3^3^-α-L-Ara*f*-(1–4)-β-D-xylotetraose (XA^3^XX, Product No. O-XA3XX), the Ara*f* substituted XOS mixture containing both XA^3^XX and 2^3^-α-L-Ara*f*-(1–4)-β-D-xylotetraose (XA^2^XX, Product No. O-XAXXMIX, notated as XA^2/3^XX in text), the Ara*f* substituted XOS 2^3^-α-L-Ara*f*-(1–4)-β-D-xylotriose (A^2^XX, Product No. O-A2XX), the Ara*f* substituted XOS 2^3^,3^3^-di-α-L-Ara*f*-(1–4)-β-D-xylotriose (A^23^XX, Product No. O-A23XX), the Ara*f* substituted XOS 2^3^,3^3^-di-α-L-Ara*f*-(1–4)-β-D-xylotetraose (XA^23^XX, Product No. O-XA23XX) and the GlcA substituted XOSs 2^3^-(4-O-Methyl-α-D-Glucuronyl)-β-D xylotetraose (XU^4m2^XX [XUXX], Product No. O-XUXX) and 2^3^-(4-O-Methyl-α-D-Glucuronyl)-β-D-xylotriose (U^4m2^XX [UXX], Product No. O-UXX), commonly referred to as aldopentauronate and aldotetrauronate, respectively, were all purchased from Megazyme (Bray, Ireland). Nomenclature utilized for naming of defined xylooligosaccharides followed the guidance of Fauré et al. [27]. As utilized above, an “U” or “A” used in the description of a branched xylooligosaccharide represents a GlcA or Ara*f* substituted xylose residue, respectively, in that specific location. When represented as “(U)” to describe a GlcA bearing branched oligoxyloside, these are referred to as aldouronates and the specific xylose bearing the GlcA is not known. An aldouronate mixture (“GXn Oligo” consisting of GlcA, U, (U)X, (U)XX….etc) was purchased from Megazyme for use as a TLC standard. All other reagents were of the highest purity available. The GH67 α-glucuronidase (Product No. E-AGUBS) from *Geobacillus stearothermophilus* and the GH43 α-L-arabinofuranosidase (AXH-D3, Product No. E-AFAM2) from *Bifidobacterium adolescentis* were also obtained from Megazyme. A xylose standard was purchased from Absolute Standards (Hamden, CT) for use in biochemical analysis.

### Cloning of the Xyl3B and Xyl3C encoding genes, protein expression and protein purification

Cloning of the *xyl3B* (GenBank accession: GU564513) and *xyl3C* (GenBank accession: GU564514) genes for protein expression were each performed using codon optimized synthetic fragments obtained from IDT (Coralville, Iowa) and Eurofins Genomics (Louisville, KY), respectively using Gibson cloning strategies [28] into pET28b + plasmid between the NcoI and XhoI restriction sites. The cloned genes encoded forms of Xyl3B (UniProt accession No. D5KJA4) and Xyl3C (UniProt accession No. D5KJA5) having truncated secretion signal sequences and a C-terminal His-Tag for affinity purification [11]. The secretion signal sequence was determined using Signal-P 5.0 [29]. Xyl3B was predicted to be processed by signal peptidase II following amino acid position 16 and Xyl3C predicted to be processed following amino acid position 21 by signal peptidase I. The expression product of Xyl3B consisted of the amino acid sequence MNNQTILI …. YFSEVIKLEHHHHHH with the N-terminal methionine replacing the native cysteine. The Xyl3C expression product consisted of MAQHLPYQ …. DPDMKKLEHHHHHH with a methionine and an alanine included at the N-terminus.

Protein expression was performed using *Escherichia coli* BL21(DE3) with autoinduction methods originally developed by Studier [30] as previously modified [31]. Cell pellets were lysed using sonication and recombinant protein was purified from the resulting lysates using Immobilized Metal Affinity Chromatography (IMAC) and a single step imidazole elution method as previously described [11]. Eluted protein was desalted using 5 ml capacity 7 kDa MWCO Zeba gel filtration spin columns (ThermoFisher Scientific, Waltham, MA) into 20 mM Tris, pH 7.4. Proteins were quantified by absorbance at 280 nm based on the ProtParam predicted extinction coefficient [32]. Protein purity was confirmed by SDS-PAGE [33] using a Bio-Rad Mini-PROTEAN system with 4–20% TGX precast gels (Bio-Rad, Hercules, Ca). Aliquots of the protein solutions were flash frozen in liquid N_2_ and stored at -80 °C.

### Chromatographic analysis for enzyme function assessment and specific activity measurements

For determination of Xyl3B and Xyl3C enzyme function, Thin Layer Chromatography (TLC) was utilized as described previously [11, 34, 35]. Briefly, glass backed silica 60 plates (Supelco Item No. 1.05626.0001, Millipore Sigma, Burlington, MA) were spotted in 1 µl aliquots of standards and reactions, and the plate was dried at approximately 55 °C prior to ascension in a 6:7:1 ratio of chloroform : acetic acid : water. At least two ascensions were performed with 30 min between each ascension for drying at 55 °C. Small scale reactions consisting of 30 mM sodium acetate pH 5.5, 0.02 mg/ml BSA and 5 mM xylooligosaccharide were equilibrated to 40 °C and initiated by addition of GH3 β-xylosidase to 100 µg/ml and allowed to react for 1 h prior to stopping the reaction by incubation at 95 °C for 15 min. Reactions for TLC that also utilized the GH67 α-glucuronidase were incubated at 60 °C for 2 h using an empirically determined GH67 dilution.

Separation of Ara*f* XOS was accomplished with High Performance Anion Exchange Chromatography (HPAEC) using a Dionex ICS 3000 system (Thermo Fisher Scientific) with amperometric detection. Separation was performed using a PA1 column with guard column at a flow rate of 1.2 ml/min and a column temperature of 30 °C. Carbohydrate separation was performed with 100 mM NaOH as mobile phase with a 0-150 mM sodium acetate gradient over 30 min. These small-scale reactions were buffered with 50 mM sodium citrate, 150 mM sodium chloride, pH 5.5, and contained 0.02 mg/ml BSA. Oligosaccharide substrate concentration was at 66.6 µg/ml and following temperature equilibration reactions were initiated by addition of enzymes. Xyl3C was utilized at 33.5 µg/ml and the dilution of the AXH-D3 arabinofuranosidase utilized was determined empirically in preliminary test reactions. Both enzymes were used at 37 °C and digestions were performed for 20 min.

For simplicity and comparison to the previous report [26], specific activity determination was performed using artificial para-nitrophenol substrates as previously described [11]. Briefly, in a 100 µl reaction containing 1 mM substrate, 30 mM sodium acetate pH 5.5 and BSA at 0.02 mg/ml, reactions were equilibrated to 40 °C and initiated by addition of enzyme. For these studies, Xyl3C and Xyl3B were utilized at 2.5 µg/ml and 1 µg/ml, respectively.

## Results and discussion

The GH3 xylosidases Xyl3B and Xyl3C from *S. baroniae* were cloned, expressed and IMAC purified. Both were obtained in quantities necessary for biochemical studies with SDS-PAGE showing protein preparations of high relative purity (Supplemental Fig. 1). Based on *in-silico* N-terminal signal peptide analysis, Xyl3B and Xyl3C are both predicted secreted enzymes. The expressed form of Xyl3B consisted of 768 amino acids, has a calculated molecular weight of 85,611 Da, a predicted isoelectric point (pI) of 5.7 and a calculated extinction coefficient of 96,150 M^− 1^ cm^− 1^ @ 280 nm. The expressed version of Xyl3C consists of 840 amino acids, has a calculated molecular weight of 93,966 Da, a predicted pI of 7.3 and a calculated extinction coefficient of 122,160 M^− 1^ cm^− 1^ @ 280 nm. Although they are both apparent GH3 xylosidases, they only share 28.5% amino acid sequence identity. Markedly, Xyl3C is predicted to be processed by signal peptidase I, while Xyl3B is predicted to be processed by signal peptidase II leaving an N-terminal cysteine (removed in the recombinant form used in this study) which indicates that Xyl3B is a likely lipoprotein which would be attached to the lipid membrane of *S. baroniae*. It is important to acknowledge that some inconsistency in the Dodd et al. [26] publication suggests the N-terminus of their Xyl3C expression product maintained the 21 amino acid signal sequence which was appended with the pET15 N-terminal fusion domain effectively adding a new N-terminal start codon and encoding an additional 20 amino acids representing a His Tag and a thrombin cleavage site. This description derives from the published cloning primers and cloning methods which is in contrast with what the authors state regarding truncation of the Signal-P detected signal peptide. If this cloning discrepancy occurred, then the Dodd et al. Xyl3C expression product would have been approx. 40 amino acids larger than the version reported in this research.

As previously reported [26], domain analysis of these GH3 xylosidases, identify the two common N-terminal and C-terminal GH3 domains, however Xyl3B contains an alignment gap when aligned with Xyl3C. This is due to a PA14 domain insertion into the middle of the C-terminal GH3 domain of Xyl3C. The PA14 domain is widely distributed [36] among bacteria and is predicted to be involved in carbohydrate binding. Dodd et al. [26] suggested that it is the PA14 domain that underlies the functional differences observed between the Xyl3C and Xyl3B. In addition, both GH3 xylosidases contain a C-terminal Fibronectin type III-like domain (FN3). These domains are highly distributed in bacteria and are commonly observed as a repeat domain in glycoside hydrolases. Biochemical studies of FN3 domains from a *Clostridium thermocellum* cellobiohydrolase supported a role for these domain in cellulose surface modification [37, 38]. It would seem unlikely for this to be the role of the FN3 domain in Xyl3B and Xyl3C.

**Fig. 2:**
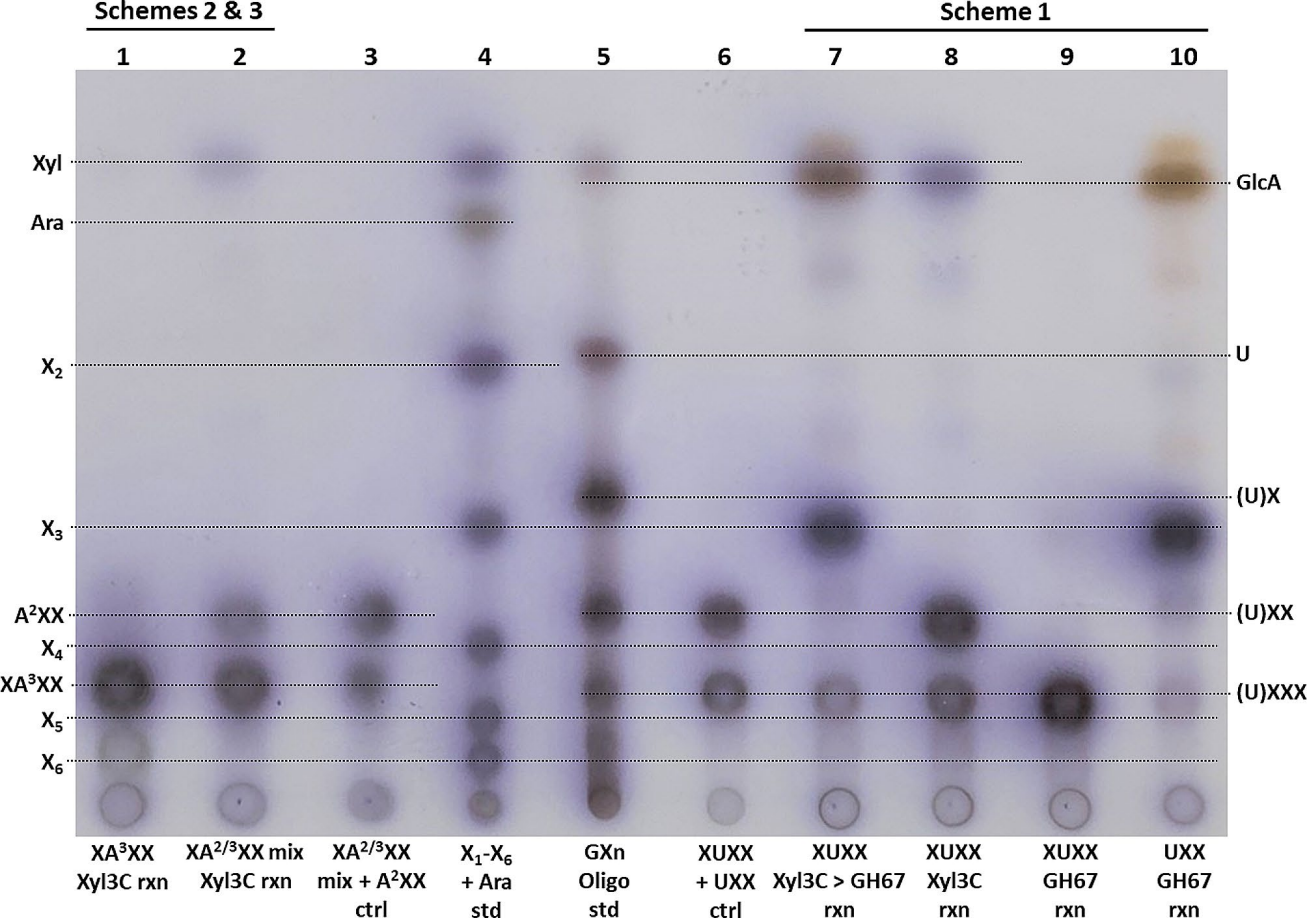
TLC analysis of Xyl3C processing of substituted XOS for confirmation of enzyme function. Lanes 3 through 6 contain standards. Lanes 1 and 2 show the Araf position dependent processing by Xyl3C (Fig. 1, Schemes 2 & 3). Lane 7 through 10 detail the selective enzymology for the use of GH67 α-glucuronidases and GH3 xylosidases similar to Xyl3C to determine the structure of the GlcA substituted oligoxyloside (Fig. 1, Scheme 1). Xyl = xylose, Ara = arabinose. Please refer to first paragraph of Materials and Methods for oligosaccharide nomenclature pertaining to figure annotation

Determination of the functional specificity of Xyl3C was performed as shown in the schemes laid out in Fig. 1, Scheme 1. Original studies of the limit hydrolysis products of GH11 endoxylanases used this two-enzyme system to show the substitution position of GlcA on the limit aldouronate, XUXX [6, 21]. Given the specificity of the GH67 α-1,2-glucuronidase [8, 18], GH3 hydrolysis of the non-reducing terminal xylose is required to prepare the aldouronate for removal of the GlcA. The TLC study displayed in Fig. 2 (lanes 7–10) shows the outcome of this analysis. The smaller aldouronate, UXX, bearing the GlcA substitution on the non-reducing terminus [6, 21] is shown to be processed by the GH67 (Fig. 2, lane 10) while the larger aldouronate XUXX is shown not to be processed (Fig. 2, lane 9). This larger aldouronate however is shown to be efficiently hydrolyzed to UXX by treatment with the GH3 Xyl3C (lane 8). When performed in series this generates xylose, GlcA and X_3_ (Fig. 2, lane 7).

The Ara*f* substituted xylotetraose XA^2^XX is structurally analogous to the aldouronate XUXX with the O2 hydroxyl of the third xylose bearing the substitution. Importantly, while the position of substitution is the same, these appendage sugars do not occupy the exact same space relative to the attached xylose as the GlcA is configured α-D and the Ara*f* α-L. In addition, the arabinose is a pentose in a furanose configuration and the GlcA an acidic hexose in a pyranose configuration thus altering how each may be accommodated by a GH3 xylosidase. However, given the bonding similarity with the O2 hydroxyl and as outlined in Fig. 1, Scheme 2, Xyl3C is anticipated to be able to remove the non-reducing terminal xylose from the O2 substituted isomer (Fig. 1, scheme 2), but not the O3 substituted isomer (Fig. 1, scheme 3). Figure 2, lanes 1 and 2 confirm that Xyl3C displays this anticipated specificity. While the XA^2^XX isomer, XA^3^XX, is readily available for studies, XA^2^XX is only available as a mixture which also contains XA^3^XX (XA^2/3^XX). Figure 2, lane 1 reveals that Xyl3C does not release the non-reducing terminal xylose when the penultimate xylose bears the substitution on the O3 position. For analysis of the O2 substituted isomer (Fig. 2, lane 2), the XA^2/3^XX mixture was treated with Xyl3C and showed release of xylose and the Ara*f* substituted XOS, A^2^XX.

**Fig. 3:**
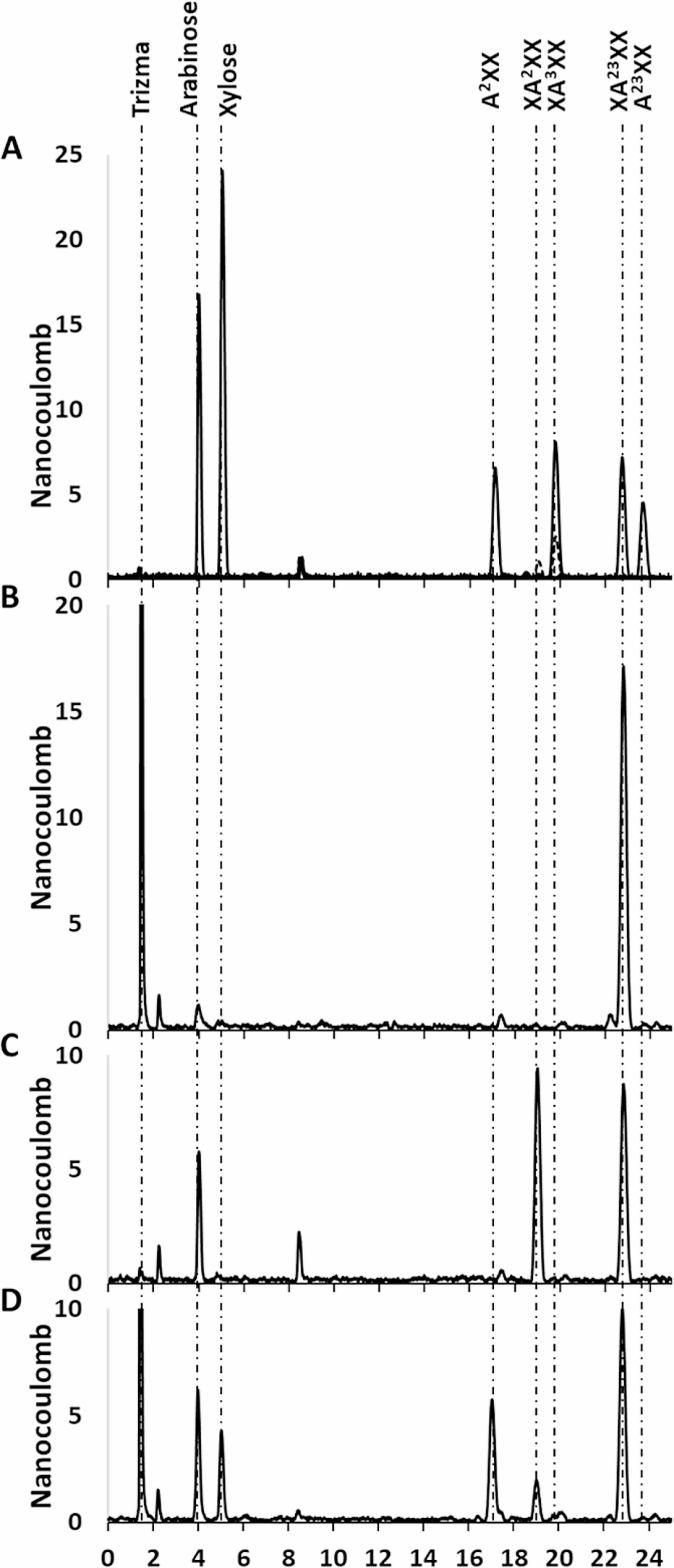
HPAEC was utilized for the resolution of the XA^2^XX and XA^3^XX isomers with Xyl3C functional verification coupled to the stepwise enzyme processing of XA^23^XX to Ara*f*, xylose and A^2^XX (Fig. 1, Scheme 4). Panel A shows elution retention times for the sugars and oligoxylosides involved in the conversion process. Importantly, the XA^2/3^XX isomer mix (dashed trace) shows both the XA^3^XX (aligned with the XA^3^XX standard) and a smaller peak at about 19 min corresponding to the XA^2^XX isomer. The peak observed at 8.4 min is determined to be a system peak

As shown in Fig. 1, scheme 4 selective processing of the XA^2^XX and XA^3^XX isomers can be further verified using the doubly Ara*f* substituted X_4_, XA^23^XX coupled with the GH43 arabinofuranosidase, AXH-D3. This enzyme removes an O3 substituted Ara*f* strictly from xylose residues that are doubly substituted, therefore leaving an O2 linked Ara*f*. To resolve the XA^2/3^XX isomers HPAEC was utilized (Fig. 3). As Fig. 3B reveals, and as anticipated from previous studies, XA^23^XX is not hydrolyzed by treatment with Xyl3C. Initial hydrolysis using the AXH-D3 arabinofuranosidase releases arabinose and generates the XA^2^XX isomer (Fig. 3C). This oligoxyloside can then be processed using Xyl3C (Fig. 3D). The net conversion generates, xylose, arabinose and the oligoxyloside A^2^XX.

From the original Dodd et. el. publication [26], data was provided indicating that Xyl3C was able to act cooperatively with a GH67 α-glucuronidase for hydrolysis of a commercial aldouronate mixture. In that study, the authors compared four GH3 glycosidases from *S. baroniae* and based on preliminary studies focused on the two most proficient xylosidases. In addition to Xyl3C this included the xylosidase Xyl3B [26]. This second xylosidase was functionally distinct from Xyl3C not allowing complete saccharification of the commercially available aldouronate preparation tested by Dodd et al. [26]. Given this distinction, we cloned Xyl3B and performed biochemical studies to verify the original observations and to confirm the observation that not all GH3 xylosidases function toward complex substituted oligoxylosides in the same manner. Supplemental Fig. 2 confirms this by showing that Xyl3B does not hydrolyze the aldouronate XUXX. While Xyl3B is not able to release this nonreducing terminal xylose, specific activity measurements (Supplemental Table 1) with pNP-Xyl shows that it has specific activity of 25.14 U/mg while Xyl3C had a specific activity of 3.13 U/mg. A difference of only eight-fold suggests that these enzymes are performing similarly.

The Xyl3C specific activity values obtained in this research however were significantly different from that reported by Dodd et al. [26] toward pNP-Xyl. In the current report, the specific activity is approximately 82-fold greater than that previously measured. We cannot account for this difference but note that if the 40 amino acid addition reported by Dodd et al. occurred, it would present a complicating factor for functional comparison. Other differences which might help account for the starkly different pNP-Xyl hydrolysis rates include the use of citrate buffer by Dodd et al. and the inclusion of BSA in this current report. Our preliminary activity assessment of Xyl3C (data not shown) indicated that citrate buffer with the 150 mM NaCl employed by Dodd et al. yielded similar activities as to our acetate condition and that BSA resulted in a significant increase in activity. Although the rate of hydrolysis of pNP-Xyl was notably different between the two Xyl3C forms, both studies yielded similarly low levels of activity for the hydrolysis of pNP-Glu. This value was about 60-fold lower than that measured in this report for the hydrolysis of pNP-Xyl. Activities of Xyl3B on both pNP-Xyl and pNP-Glu are approx. 2-fold lower than the measurements reported by Dodd et al. with activity on the xylose analog being about 50–60 fold greater than pNP-Glu. The two-fold difference between the reports is not surprising. For the purposes of this report, Xyl3B studies only sought to contrast the unique functional features of Xyl3C. Both Xyl3B and Xyl3C had no significant activity toward pNP-Ara*p*.

## Conclusions

The data presented in this report clarifies the functional specificity of the Xyl3C xylosidase from *S. baroniae*. Xyl3C can be utilized for Ara*f* and GlcA branched xylooligosaccharide structure determination. For laboratories involved in this field of research Xyl3C will be easy to obtain and will provide confident xylooligosaccharide structure determination analysis when utilized as described in this report.

## Limitation

While this report addresses several aspects of the original Dodd et al. publication [26] it primarily only sought to determine the fine functional specificity characteristics of the *S. baroniae* Xyl3C xylosidase. In consideration of this, the protein was of limited purity and methodical comparison of the enzyme biochemistry was beyond the scope of this work.

### Electronic supplementary material

Below is the link to the electronic supplementary material.

Supplementary Material 1

Supplementary Material 2

## Data Availability

Data and materials are available from the corresponding author upon request.
