# Supplementary material for: Use of xylosidase 3C from Segatella baroniae to discriminate xylan non-reducing terminus substitution characteristics
Source: BMC Res Notes. 2024 Jun 24;17:175. doi: 10.1186/s13104-024-06835-3 (PMC11197168; doi:10.1186/s13104-024-06835-3)
Supplement: Supplementary file 1 — Supplementary Material 1 [file 13104_2024_6835_MOESM1_ESM.pptx]

## Slide 1
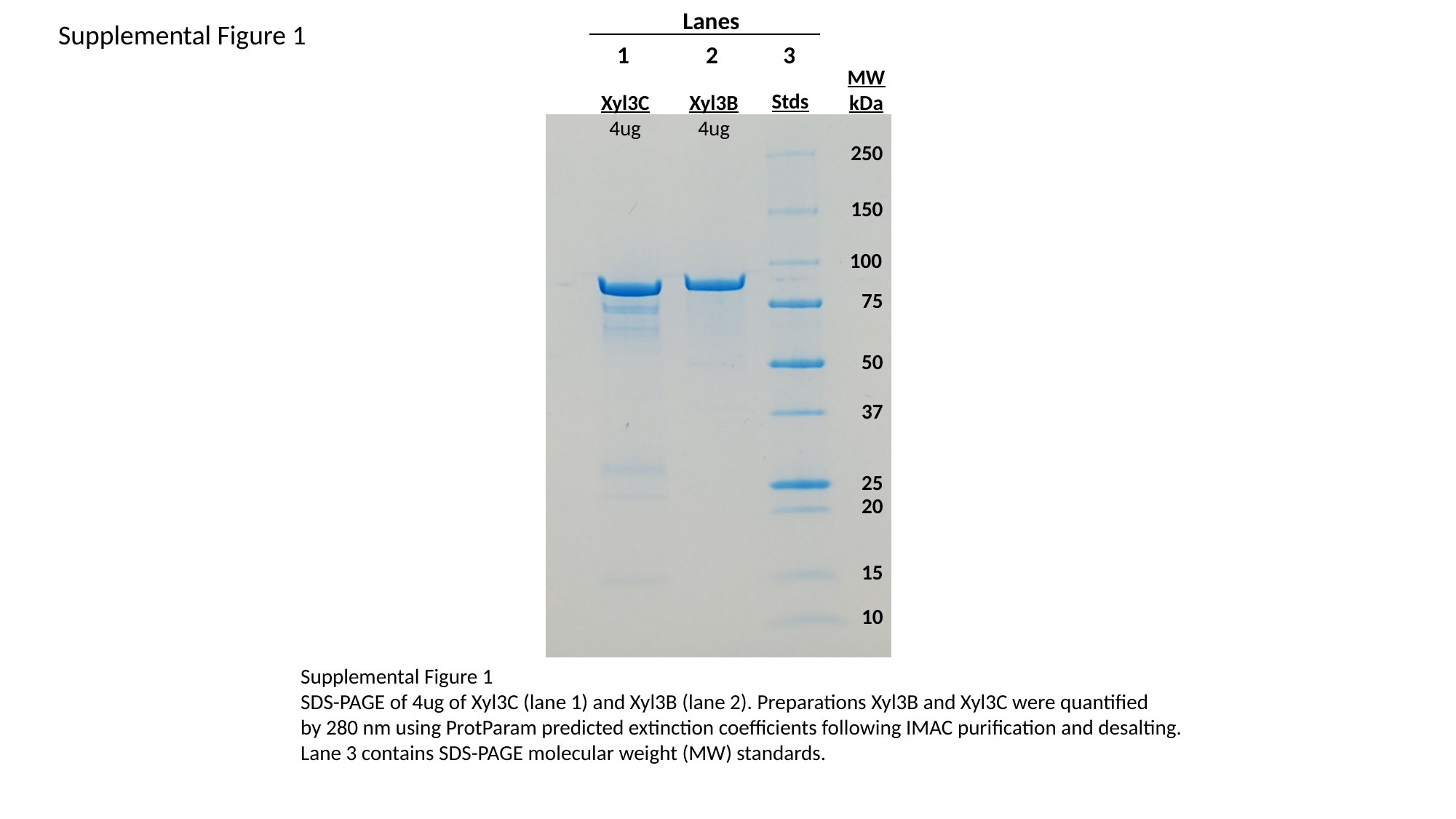

Lanes
Supplemental Figure 1
1
2
3
MW
kDa
Stds
Xyl3B
4ug
Xyl3C
4ug
250
150
100
75
50
37
25
20
15
10
Supplemental Figure 1
SDS-PAGE of 4ug of Xyl3C (lane 1) and Xyl3B (lane 2). Preparations Xyl3B and Xyl3C were quantified
by 280 nm using ProtParam predicted extinction coefficients following IMAC purification and desalting.
Lane 3 contains SDS-PAGE molecular weight (MW) standards.

## Slide 2
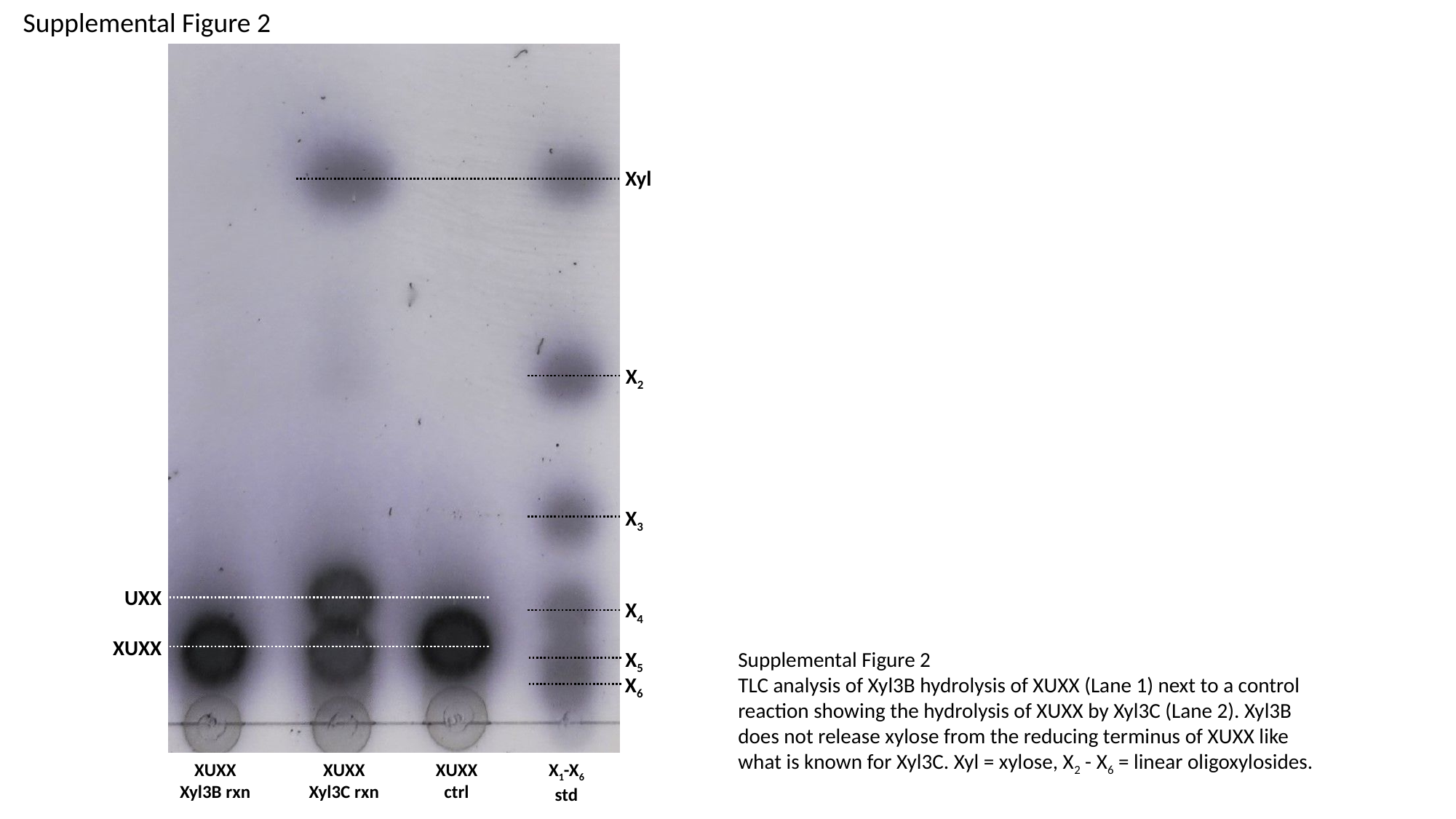

Supplemental Figure 2
Xyl
X2
X3
UXX
X4
XUXX
X5
Supplemental Figure 2
TLC analysis of Xyl3B hydrolysis of XUXX (Lane 1) next to a control
reaction showing the hydrolysis of XUXX by Xyl3C (Lane 2). Xyl3B
does not release xylose from the reducing terminus of XUXX like
what is known for Xyl3C. Xyl = xylose, X2 - X6 = linear oligoxylosides.
X6
XUXX
Xyl3B rxn
XUXX
Xyl3C rxn
XUXX
ctrl
X1-X6
std
