# Supplementary material for: Use of xylosidase 3C from Segatella baroniae to discriminate xylan non-reducing terminus substitution characteristics
Source: BMC Res Notes. 2024 Jun 24;17:175. doi: 10.1186/s13104-024-06835-3 (PMC11197168; doi:10.1186/s13104-024-06835-3)
Supplement: Supplementary file 2 — Supplementary Material 2 [file 13104_2024_6835_MOESM2_ESM.docx]

**Supplementary Table 1**

**Specific Activity Measurements for Xyl3B and Xyl3C**

| **Substrate** | **Specific Activity Measurements ^1^** | |
| --- | --- | --- |
|  | **Xyn3B** | **Xyn3C** |
| pNP-Xyl | 25.14 ± 0.44 | 3.13 ± 0.31 |
| pNP-Ara*p* | ND^2^ | ND^2^ |
| pNP-Glu | 0.41 ± 0.01 | 0.05 ± 0.01 |

^1^ Specific activity is reported as Units/mg protein where 1 Unit equals 1 umole pNP

released per minutes of reaction time.

^2^  None detected.
